# A unique case of late-onset CIPO caused by a missense mutation in the long isoform of *FLNA*

**DOI:** 10.3389/fgene.2025.1611614

**Published:** 2025-08-08

**Authors:** Ilaria D’Amato, Enrico Ganguzza, Guido Basilisco, Alessia Strippoli, Erika Salvi, Elkadia Mehmeti, Federica Chiappori, Grazia Devigili, Maurizio Vecchi, Giuseppe Lauria, Margherita Marchi

**Affiliations:** ^1^ Neuroalgology Unit, Fondazione IRCCS Istituto Neurologico Carlo Besta, Milan, Italy; ^2^ Gastroenterology and Endoscopic Unit, Fondazione IRCCS Ca’ Granda Ospedale Maggiore Policlinico, Milan, Italy; ^3^ Department of Pathophysiology and Transplantation, Università degli Studi di Milano, Milan, Italy; ^4^ Consiglio Nazionale delle Ricerche, Istituto di Tecnologie Biomediche (CNR-ITB), Segrate, Italy; ^5^ Movement Disorders Unit, Fondazione IRCCS Istituto Neurologico Carlo Besta, Milan, Italy; ^6^ Department of Medical Biotechnology and Translational Medicine, Università degli Studi di Milano, Milan, Italy

**Keywords:** chronic intestinal pseudo-obstruction, CIPO, FLNA, filaminopathies, whole exome sequencing

## Abstract

Mutations in the filamin A (FLNA) gene cause a broad range of disorders, affecting musculoskeletal, nervous, vascular, and gastrointestinal systems, collectively known as filaminopathies. In contrast to previously described mutations in the long isoform of *FLNA*, which alter the reading frame and lead to loss of Filamin A expression resulting in congenital short bowel syndrome or chronic intestinal pseudo-obstruction in pediatric patients, here we present the clinical and genetic features of an adult patient with chronic intestinal pseudo-obstruction in whom whole exome sequencing revealed a novel missense mutation (p.Gly19Val) in *FLNA* gene. The onset of symptoms was at 31 years old when he began experiencing constipation, vomiting, and weight loss. Segregation analysis showed that the p.Gly19Val mutation was inherited from the heterozygous unaffected mother and was absent in the healthy brother and father, consistent with X-linked recessive inheritance. The mutation was localized in the N-terminus of the *FLNA* long isoform, a critical region for smooth muscle contractility and intestinal motility. Structural modeling of the mutant Filamin A suggested that the p.Gly19Val substitution alters the local protein folding and may interfere with the protein ability to cross-link actin filaments, potentially impairing cytoskeletal dynamics in visceral smooth muscle cells. Our study broadens the phenotypic spectrum of filaminopathies and deepens the understanding of the genetic mechanisms underlying chronic intestinal pseudo-obstruction in adults.

## 1 Introduction

Chronic intestinal pseudo-obstruction (CIPO) is a rare and disabling syndrome, characterized by recurrent symptoms of intestinal obstruction in the absence of any mechanical occlusive lesion, that can arise at any age (Downes et al., 2018; Basilisco et al., 2024). CIPO results from the damage of gut wall intrinsic neuronal, muscular, or mesenchymal structures or its extrinsic neuronal control, leading to chronic impairment of tonic and propulsive motor functions in one or more segments of the gut (Downes et al., 2018; Basilisco et al., 2024). This condition can be associated with systemic diseases or be genetically determined with autosomal dominant, recessive, and X-linked inheritance patterns (Downes et al., 2018; Bianco et al., 2022; Basilisco et al., 2024). The X-linked form of CIPO (CIIPX; OMIM #300048) (McKusick- Nathans Institute of Genetic Medicine, 2023) is caused by mutations in the filamin A (*FLNA*) gene. FLNA is a cytoplasmic actin-binding protein involved in cell motility, migration, and maintenance of cytoskeletal integrity (Zada et al., 2023). Mutations of the *FLNA* gene cause a spectrum of disorders, called filaminopathies, characterized by dysmorphic features, abnormal neuronal migration, vascular and cardiac defects, and intestinal dysmotility leading to CIPO and congenital short bowel syndrome (CSBS) (OMIM #615237) (Popowicz et al., 2006; Wade et al., 2020; McKusick- Nathans Institute of Genetic Medicine, 2023). Differential expression of *FLNA* transcripts accounts for some of the phenotypic variability observed in filaminopathies. Loss-of-function mutations in males, who are constitutively hemizygous for these alleles, are commonly lethal. Genetic studies on *FLNA* variants associated with CIIPX and CSBS revealed that rare surviving male patients, obligatory carriers of *FLNA* variants, have loss-of-function mutations localized at the 5′ terminus of the longest isoform. The impairment of the long isoform mainly compromises the intestinal expression of Filamin A, resulting in impaired smooth muscle contractility and reduced intestinal development (van der Werf et al., 2013; Jenkins et al., 2018; Zada et al., 2023). In 1996 Auricchio et al. identified a genomic locus associated with CIPO, providing the first evidence of *FLNA* involvement in the pathogenesis of the disease (Auricchio et al., 1996). Subsequently, in 2007, Gargiulo et al. identified a specific genetic mutation within the same family: a 2-base pair deletion in exon 2 of the *FLNA* gene (Gargiulo et al., 2007). Segregation analysis confirmed that unaffected carrier mothers were heterozygous for this deletion. Since then, other studies have reported cases of CIPO related to filaminopathies, all involving pediatric male patients (FitzPatrick et al., 1997; Guerrini et al., 2004; Hehr, 2006; Clayton-Smith et al., 2009; Kapur et al., 2010; Oda et al., 2016; Berrou et al., 2017; Jenkins et al., 2018). Herein, we report the first case of an adult patient with CIPO associated with a novel missense mutation (p.Gly19Val) in the *FLNA* gene. The genetic variant was located in the long isoform of the gene (NM_001110556.2), between the first (ATG^+1^) and the second starting site (ATG^+82^), a region known to be essential for smooth muscle contractility and intestinal motility (Jenkins et al., 2018; Zada et al., 2023).

## 2 Materials and methods

### 2.1 Subjects

The index case of this study was evaluated at Gastroenterology and Endoscopic Unit, Fondazione IRCCS Ca’ Granda Ospedale Maggiore Policlinico (Milan, Italy). The proband and his family were enrolled for a collaborative study and underwent genetic investigation at the Fondazione IRCCS Istituto Neurologico Carlo Besta (Milan, Italy). The participants in this study, provided written informed consent following the ethical recommendations at Besta Neurological Institute. Phenotyping was performed by experienced gastroenterologists and genetic analysis conducted by a senior geneticist.

### 2.2 Genetic analysis

#### 2.2.1 Genomic DNA extraction

Peripheral blood was collected from subject II/2, and saliva samples from I/1, I/2, and II/1 (Supplementary Figure S1) in Oragene tubes (DNA Genotek Ink, Stittsville, Canada). Genomic DNA was extracted from whole blood using the Puregene Blood Kit (Qiagen, Hilden, Germany), according to the manufacturer’s instructions; gDNA from saliva specimens was extracted using the prepIT.L2P reagent (DNA Genotek Ink) according to the manufacturer’s guidelines.

#### 2.2.2 Whole-exome sequencing and data analysis

Whole-exome sequencing (WES) was performed to find possible variants of interest in genes related to CIPO.

To capture the exomes Agilent SureSelect Human All Exon V7 (Agilent Technologies, Santa Clara, California, US) was used, followed by paired-end 150bp sequencing on an Illumina HiSeq2000 sequencing platform (Illumina, San Diego, California, US). Raw sequencing files underwent quality control using FastQC (Andrews S, 2010). The exome-captured sequencing reads were aligned to the NCBI human reference genome GRCh37 (hg19) with Burrows-Wheeler Aligner MEM (BWA MEM v0.7.17) (Li and Durbin, 2009). Aligned reads were then processed to mark duplicates using Picard v2.4.1 (Broad Institute, 2024). Variant calling was performed using the HaplotypeCaller of the Genome Analysis Toolkit (GATK v4.1.9) (Van der Auwera and O’Connor, 2020). Finally, single-nucleotide variants identified by GATK were annotated using SnpEFF v5.0 (Cingolani et al., 2012).

To identify the variants of interest (VOIs), a multistep filtering process was applied based on the following criteria: a minimum coverage of 20X, an allelic balance greater than 25%, and the exclusion of captured intergenic and UTR variants, non-splice-related intronic variants, and synonymous variants. Further refinement was performed using the GnomAD database (Chen et al., 2024), applying a frequency threshold of less than 0.5%. All private variants, absent from the consulted datasets, were considered.

Predictions on variant deleteriousness are based on SIFT, PolyPhen-2, and GERP scores obtained using SnpEFF v5.0 (Cingolani et al., 2012). Based on bioinformatics predictions of variant deleteriousness, we conducted manual searches in PubMed (Bethesda, 1946) to select genes, evaluating them based on available information regarding their expression and function in the intestine.

#### 2.2.3 Polymerase chain reaction (PCR) and sanger sequencing

Exon 2 of the *FLNA* gene was amplified using Go Taq G2 (Promega Corporation, Madison, Wisconsin, US) according to the manufacturer’s protocol using a Touchdown thermal protocol from 65°C to 55°C in the annealing step (Supplementary Tables S1, S2). Amplicons were purified with Illustra ExoProStar1-Step (GE Healthcare, General Electric Company, Schenectady, New York, US) and bidirectionally sequenced using BigDye Terminator v3.1 Cycle Sequencing Kit (Thermo Fisher Scientific, Waltham, Massachusetts, US) on a 3130xL Genetic Analyzer (Thermo Fisher Scientific). The electropherograms were analyzed using Codon Code Aligner software.

### 2.3 Phylogenetic analysis

To investigate the physio-chemical perturbation in the surrounding region, we analyzed the characteristics of adjacent residues and compared them across different animal species to assess the phylogenetic conservation of the region, using the UniProt database (Bateman et al., 2025). We compared species selected based on their feeding habits to minimize the variability in filamin A sequence conservation that could arise from differences in feeding behavior and associated intestinal structure and function: ruminants (bovine F1N169), omnivores/facultative carnivores (dog A0A8C0TBZ3, human P21333, primates H2PX81-A0A2K6DQ90-G3QNL9, bear A0A8M1FHA2), rodents (rat A6KRS3, mouse Q8BTM8, guinea pig A0A286XNP9), and obligate carnivores (felines A0A8C8X617-A0A6P6IPA1) (Supplementary Table S3).

### 2.4 Structural evaluation

To evaluate the effect of p.Gly19Val substitution on protein structure, we predict the secondary structure and the disorder region by PsiPred 4.0 (Jones, 1999) and DisoPred 3 (Jones et al., 2015). Moreover, due to the unavailability of an experimentally resolved filamin A structure, we modeled the mutant and the wild type by RosettaCM (Song et al., 2013)

## 3 Results

### 3.1 Clinical description

**FIGURE 1:**
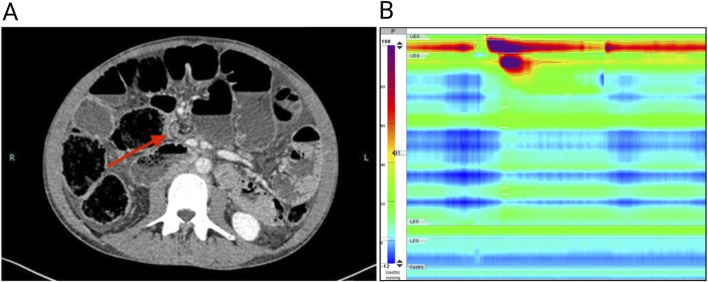
**(A)** Computed tomography of the abdomen showing a grossly dilated (6 cm diameter) small intestine; the arrow shows a jump in caliber close to the ileocecal valve. **(B)** High-resolution esophageal manometry shows the absence of esophageal contractility after wet swallows.

A 36-year-old Caucasian man presenting with abdominal pain, nausea, vomiting and constipation was admitted to our hospital with suspected ileal obstruction. He was underweight (body mass index of 17.3 kg/m^2^) with a gas-distended abdomen. Neurological examination was normal. Cardiological examination showed preserved systolic and diastolic functions, mild mitral valve prolapse with minimal regurgitation, and diffuse pericardial effusion, which was hemodynamically insignificant and attributed to malnutrition. He also had an untreated anxiety-depressive syndrome. Laboratory tests, including complete blood count, C-reactive protein, serum electrolytes (sodium, potassium, calcium, phosphorus), thyroid-stimulating hormone, immunoglobulin A, and anti-transglutaminase antibodies, were all within normal ranges. The patient was placed on a fasting regimen and a nasogastric tube was inserted for aspiration. Total parenteral nutrition and antibiotic therapy for bacterial overgrowth in the small intestine were started. Abdominal computed tomography revealed distention of the esophagus, stomach, and small intestine. Small bowel dilatation was severe (6 cm), with a change in caliber close to the ileocecal valve (Figure 1A). The bladder was not distended, and no vascular abnormalities were detected. Suspecting bowel obstruction due to adhesions, the patient underwent exploratory video-laparoscopy with adhesiolysis between the cecum and terminal ileum, but no mechanical obstruction was found and no specific intervention for the small bowel dilatation was performed.

The patient’s medical history included a monoclonal gammopathy of undetermined significance and a thalassemia trait. He had two episodes of uncomplicated pericarditis, both treated conservatively at the age of 26 and 27. His surgical history included the correction of inguinal and umbilical hernias at the age of 23 and the derotation of a transverse colon volvulus, which was attributed to intestinal adhesions, at the age of 33. Gastrointestinal symptoms started at the age of 31 when he began to experience decreased bowel movement frequency and occasional vomiting, accompanied by a 30 kg weight loss over the subsequent 5 years. Gastroenterological investigations performed 1 year before the admission included an esophagogastroduodenoscopy that revealed Barrett’s esophagus; pantoprazole 40 mg daily was prescribed to manage gastroesophageal reflux symptoms. Ileocolonoscopy revealed no macroscopic abnormalities and ileal biopsies showed chronic nonspecific ileitis. Fecal calprotectin levels were normal. Abdominal magnetic resonance imaging demonstrated uniform distention of the jejunal and ileal loops, without pathologic wall thickening or hyperenhancement. Notably, the ileum was distended with fecaloid material, and the adjacent mesentery appeared fibrotic.

Given the chronic signs and symptoms of bowel obstruction and the absence of mechanical occluding lesions, the patient underwent a diagnostic workup for CIPO. Secondary causes of CIPO including mitochondrial and neurological diseases, amyloidosis, autoimmune and connective tissue disorders, infectious disorders such as Lyme disease and Strongyloides stercoralis infections or an underlying neoplasia were excluded (Basilisco et al., 2024). Dysautonomic symptoms were not present. High-resolution esophageal manometry revealed an absence of esophageal contractility (Figure 1B), suggesting a myopathic form of CIPO. The peristaltic wave after wet swallows was absent, with a severe reduction in distal contractile integral (54 mmHg/s/cm; reference value 450–8,000 mmHg/s/cm). The resting pressure of the lower esophageal sphincter was normal (12 mmHg; reference value 9–51 mmHg), as was the median integrated relaxation index of (12 mmHg; reference value < 15 mmHg). Uroflowmetry was normal. Based on these findings, a provisional diagnosis of CIPO due to an underlying myopathy of unknown cause was made. The family history was negative for gastrointestinal, neural, vascular or cardiac, disorders possibly related to hereditary myopathies or neuropathies. The patient underwent genetic screening through whole exome sequencing. Asymptomatic family members were included for subsequent segregation analysis through Sanger sequencing (genetic testing reported in the Supplementary material and methods). After diagnosis, the patient was followed by a nutrition center to continue parenteral nutrition and remained clinically stable for 3 years. At age 39, he was urgently admitted to another hospital where a decompressive cecostomy was performed for untreatable occlusive symptoms. The surgery was complicated by colonic intussusception through the cecostomy site, necessitating an ileo-colic resection with ileo-transverse anastomosis. Postoperatively, the patient developed a bowel perforation and anastomotic dehiscence, prompting a second ileocolic resection and additional surgery to manage a hemoperitoneum. Despite the initial improvement during a 2-month intensive care unit stay, the patient died in a rehabilitation facility.

### 3.2 Genetic analysis

**FIGURE 2:**
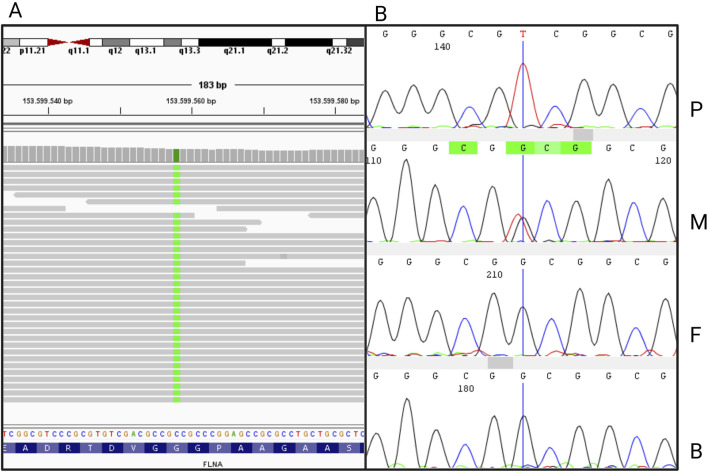
**(A)** Integrative Genomics Viewer (IGV) visualization of the nucleotide variant c.56G>T in *FLNA*, generated from the bam file obtained from whole exome sequencing. **(B)** Sanger sequence electropherograms of *FLNA* exon 2 from the proband (P), the mother (M), the father (F), and the brother (B).

Exome sequencing gained 20X reading depth for 83.6% of the analyzed bases. Genetic analysis revealed the c.56G>T hemizygous variant in the *FLNA* gene (NM_001110556.2) in the patient (Figure 2A). Sanger sequencing confirmed the variant in the patient and its heterozygous presence in the healthy mother. The variant was absent in the healthy father and brother (Figure 2B; Supplementary Figure S1). The variant c.56G>T in *FLNA* is a novel genetic mutation, never reported in the scientific literature or public genetic databases (PubMed, GnomAD, ClinVar) (Bethesda (MD): National Library of Medicine (US), 1946; Landrum et al., 2014; Chen et al., 2024). The nucleotide substitution is translated into a missense variant substituting the residue Glycine with a Valine in position 19 (p.Gly19Val). According to the American College of Medical Genetics and Genomics (ACMG), it is classified as a variant of uncertain significance. According to clinical and genetic findings, a final diagnosis of CIPO due to a novel missense mutation in the *FLNA* gene was made.

### 3.3 Comparative protein sequence analysis

The missense mutation p.Gly19Val localizes in the actin-binding domain of the protein, potentially affecting the ability of filamin A to bind actin filaments properly (Iwamoto et al., 2018). This domain is highly conserved among species, particularly in those with feeding habits and related intestinal structure and function similar to humans, such as omnivores, facultative carnivores, and obligate carnivores (Supplementary Figure S2). Not surprisingly, the more similar was the feeding habit, the higher conservation is observed in the filamin N-terminus. In particular, the glycine residue in position 19 resulted highly conserved among omnivores and facultative carnivores, conferring specific features in terms of physical and structural properties (Supplementary Figures S3–S7; Supplementary Table S3).

### 3.4 Protein structural analysis

**FIGURE 3:**
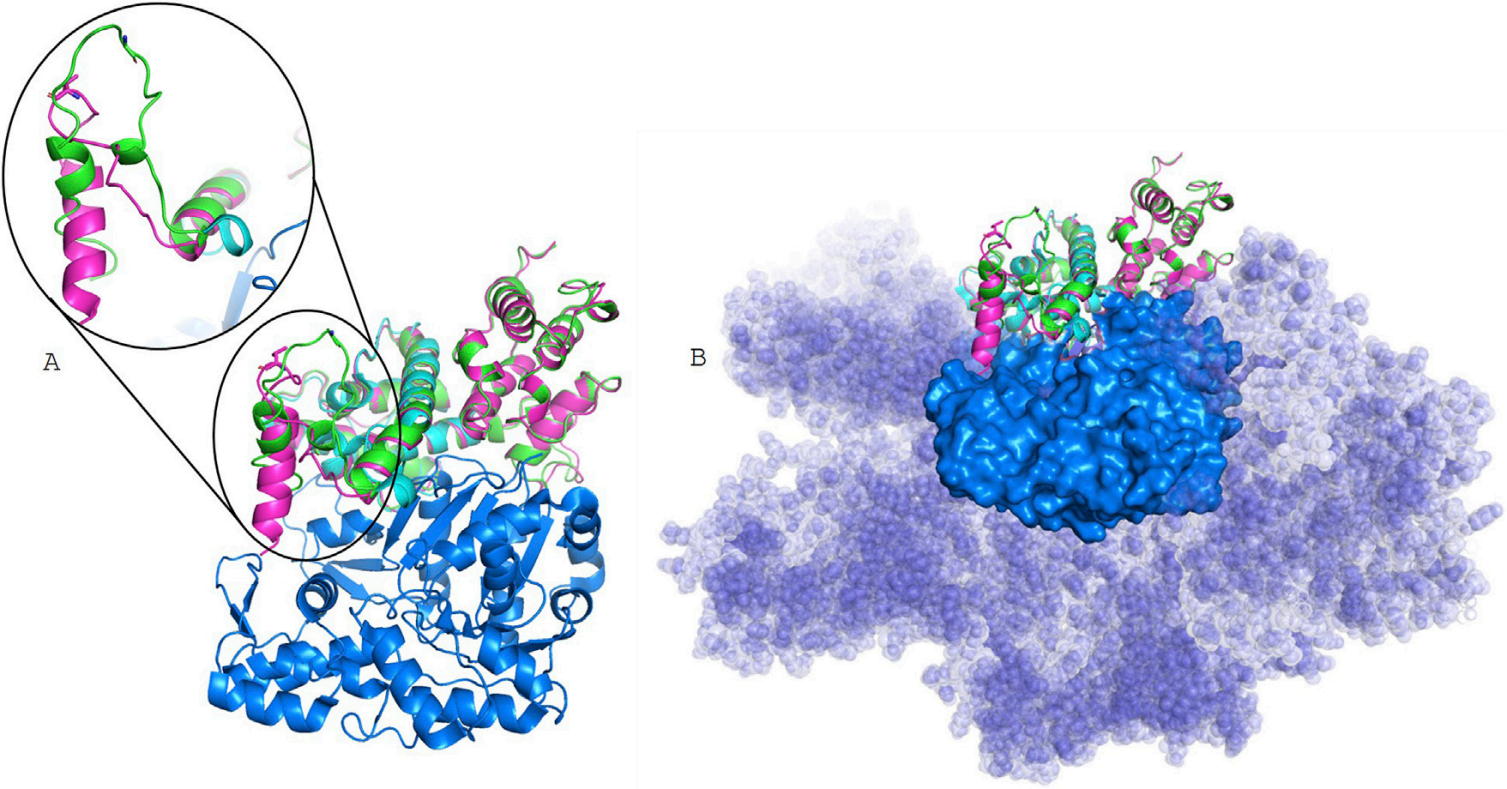
Structural models. the Filamin A experimentally resolved structure (light cyan) bound to actin filament (6D8C), represented as blue surface; in green Filamin A WT model, and in pink Filamin A Gly19Val model, in stick is shown residue 19. In **(A)**, models are superposed to the monomer extracted from 6D8C, while in **(B)** is evidenced the surface of the whole actin filament.

Secondary structure prediction algorithms classify the N-terminal 30 residues as an intrinsically disordered region, lacking a stable tertiary conformation under physiological conditions. Consistently, in the available experimental cryo-EM structure (PDB ID: 6D8C), the first 28 residues are unresolved, while residues from position 39 onward fold into an α-helix. No relevant differences were predicted between Gly19Val mutant and the WT Filamin A secondary structures (Supplementary Figure S8). Also, 3D models were obtained for both Filamin A WT and mutant (Figure 3) by RoseTTAFold. In contrast to secondary structure prediction, the first 30 residues partially fold into alpha helixes, especially for the Gly19Val mutant. This different folding allows the localization of the helix in a pocket of actin surface, without interfering with actin filament binding.

## 4 Discussion

We report the first case of a novel missense mutation (p.Gly19Val) in *FLNA* gene. The p.Gly19Val aminoacidic substitution represents the first missense variant associated with the X-linked form of CIPO. Being located 63 bp upstream of the short-isoform starting site (ATG^+82^), the variant is translated only in the long isoform of filamin A, that is predominantly expressed in the smooth muscle of the intestinal wall. Filamin A, encoded by the *FLNA* gene on Xq28, is a large actin-binding protein forming non-covalent dimers, each consisting of 2,647 amino acids. It features an actin-binding domain (ABD), 23 repeats, and a dimerization site at repeat R24. Filamin cross-links filamentous actin (F-actin), providing cytoskeletal stability, and interacts with over 100 proteins, including integrins, signaling mediators, and transmembrane receptors. These interactions regulate key processes like cell morphology, adhesion, migration, differentiation, and mechanical force sensing, essential for the integrity and function of tissues such as brain, bone, and muscle (Popowicz et al., 2006; Onoprishvili et al., 2008; Truong et al., 2015; Iwamoto et al., 2018). Mutations in the X-linked *FLNA* gene are associated with a range of conditions collectively termed filaminopathies (Wade et al., 2020), encompassing cardiac valvular dysplasia, fronto-metaphyseal dysplasia, periventricular nodular heterotopia, Melnick-Needles syndrome, otopalatodigital syndromes, terminal bone dysplasia, congenital short bowel syndrome, and intestinal pseudo-obstruction (McKusick- Nathans Institute of Genetic Medicine, 2023). Null mutations, such as nonsense or frameshift, are typically embryonically lethal in males reflecting its crucial role, whereas in heterozygous females they cause periventricular nodular heterotopia, often accompanied by cardiovascular defects. Mutations in patients with periventricular nodular heterotopia generally result in FLNA loss of function, with rare surviving males exhibiting partial loss-of-function variants and females carrying more severe mutations (Sheen, 2001). Specific missense mutations, likely having a gain-of-function effect, are linked to otopalatodigital syndromes (Iwamoto et al., 2018). Mutations specifically affecting the long isoform of filamin A are associated with congenital short bowel syndrome without neurological features (van der Werf et al., 2013; Wang et al., 2021). Since the first evidence by Auricchio and collaborators in 1996 (Auricchio et al., 1996), 31 cases of CIPO linked to filaminopathies have been reported, all in male patients (FitzPatrick et al., 1997; Guerrini et al., 2004; Hehr, 2006; Clayton-Smith et al., 2009; Kapur et al., 2010; Oda et al., 2016; Berrou et al., 2017; Jenkins et al., 2018). Additionally, two cases of isolated congenital short bowel syndrome carrying mutations affecting the long isoform of *FLNA* were reported by Van Der Werf and Wang (van der Werf et al., 2013; Wang et al., 2021). The pleiotropic effects of *FLNA* mutations are linked to the expression of two filamin A isoforms, which differ by 28 residues at the N-terminus, with translation initiation at ATG+1 for the long isoform (*FLNA* ^+1^) and ATG+82 for the short isoform (*FLNA* ^+82^) (Jenkins et al., 2018). Expression studies demonstrated that these isoforms play a key role in determining the clinical phenotype.

**FIGURE 4:**
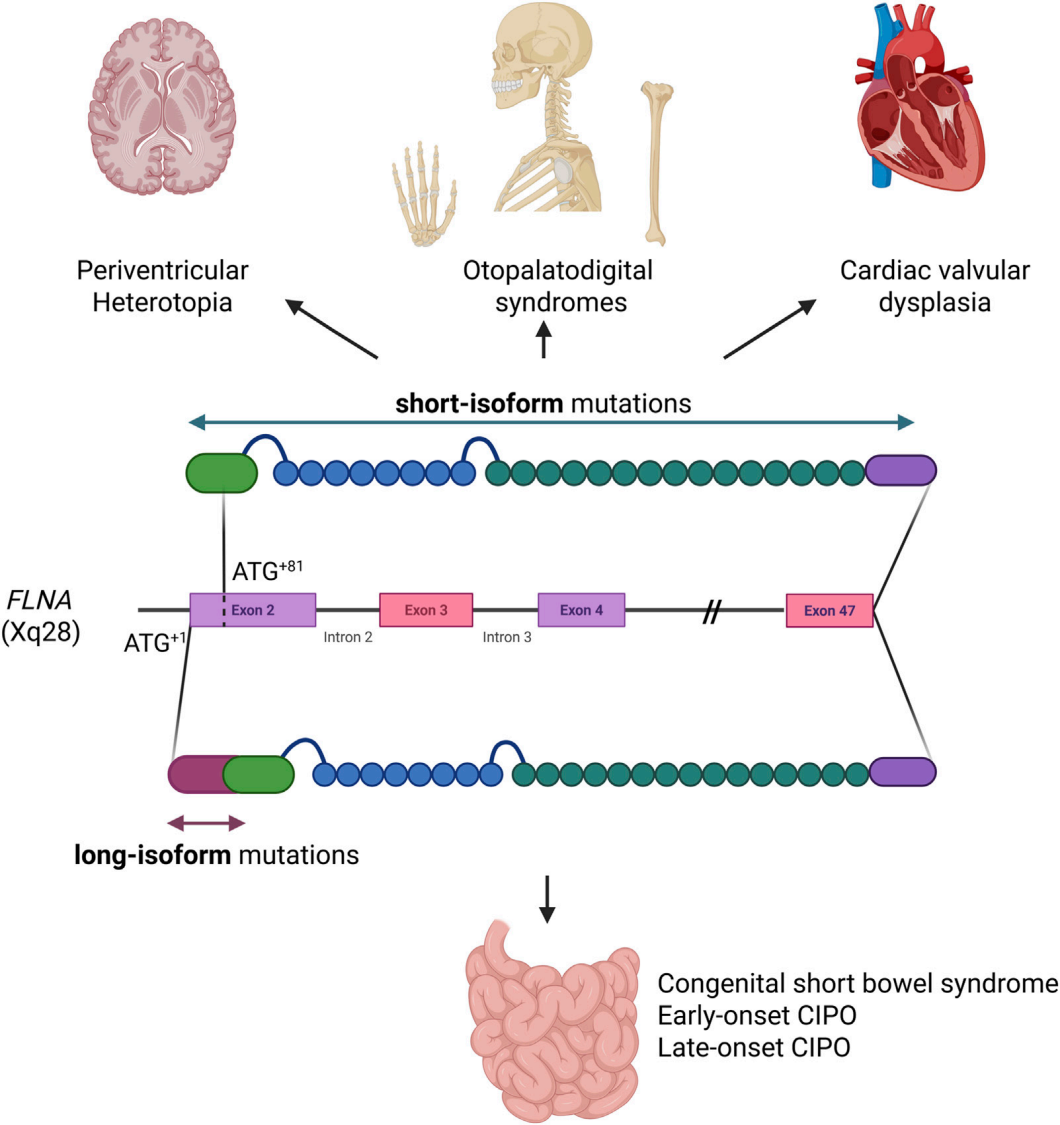
Graphical representation of the two filamin A isoforms. Disorders linked to the widely expressed short isoform are shown above, while disorders due to mutations affecting exclusively the long isoform, restricted to the small intestine, are shown below (*Created in BioRender (2025)* https://BioRender.com/n13j591)

The long isoform of filamin A (*FLNA* ^+1^) is predominantly expressed in intestinal smooth muscle and is required for intestinal development and motility. Its deficiency is associated with CIPO. In contrast, the short isoform (*FLNA* ^+82^) is more widely expressed in other tissues, particularly the brain, and its deficiency has been associated with periventricular nodular heterotopia (Zada et al., 2023). In patients with combined CIPO, periventricular nodular heterotopia, and cardiovascular changes, mutations affecting both isoforms of *FLNA* contribute to the complexity of the phenotype (Jenkins et al., 2018) (Figure 4). Similar to a case previously described (Jenkins et al., 2018), in which a c.18_19 deletion affected the long isoform of *FLNA*, our patient exhibited an exclusively gastrointestinal phenotype, without the neurological and cardiac abnormalities typically seen in filaminopathies involving both isoforms or the short isoform. However, in contrast to the case reported by Jenkins and collaborators (Jenkins et al., 2018), and other cases of CIPO associated with *FLNA* mutations, which predominantly occur during the childhood, our patient presented a late-onset of the syndrome. This difference may reflect the impact of the mutation type, with microdeletions causing premature protein truncation and complete loss-of-function likely resulting in more severe phenotypes, whereas missense mutations, like p.Gly19Val, partially preserving filamin A function and leading to a milder, late-onset presentation. The N-terminus region where the amino acidic substitution p.Gly19Val is located, is present only in the long isoform, predominantly expressed in the intestine, where it has been demonstrated to be essential for human small bowel function and development. The high degree of conservation suggests that the p.Gly19Val mutation, substituting the glycine with valine at position 19, could significantly alter the physical and structural properties of the protein, potentially impairing its function and contributing to pathogenicity. The physiochemical characteristics further support its pathogenic effects of their substitution, as glycine (Gly) and valine (Val) differ in size, flexibility, and hydrophobicity. Glycine is the smallest amino acid with a flexible hydrogen side chain, whereas valine has a bulkier isopropyl side chain, introducing rigidity. Glycine is more soluble, whereas valine is hydrophobic, which may disrupt protein interactions.

Structural analysis suggests that the FLNA variant p.Gly19Val does not directly interfere with F-actin polymerization. However, one key functions of filamin A is to organize and stabilize orthogonal actin networks by bridging filaments at flexible angles. The altered folding of the mutated protein may disrupt the spatial conformation necessary for cross-linking adjacent actin filaments. Such a structural alteration could impair its role in maintaining cytoskeletal architecture and dynamics, potentially inducing conformational distortions that compromise its biological function and contribute to disease pathogenesis. This hypothesis, however, cannot currently be tested, as high-resolution structures of the full FLNA-actin filaments complex are not yet available in public structural databases. Nevertheless, it is highly consistent with the clinical findings: esophageal manometry in our patient revealed a severe impairment of muscle contractility, strongly suggestive of a myopathic form of CIPO. Similar impairment of esophageal motility was described in pediatric and adult patients with visceral myopathy associated with ACTG2 gene mutations (Kocoshis et al., 2019; Topa et al., 2023). In line with these findings, Kapur and collaborators previously reported the histological analysis of the intestines of CIPO patients harboring *FLNA* mutations, revealing significant muscle abnormalities (Kapur et al., 2010). The small intestine was shorter than normal, malrotated, and exhibited disrupted architecture of the muscularis propria, with diffuse abnormal layering of smooth muscle. No alterations of the enteric nervous system were reported. Moreover, Zada and collaborators studied a transgenic zebrafish model expressing only the short *FLNA* isoform. In this model, the small intestine was shortened, the smooth muscle layering was abnormal, and neuronal organization remained unaffected, further supporting a myopathic origin for CIPO associated with the absence of the long isoform of the *FLNA* (Zada et al., 2023). However, it should be noted that, in contrast with these observations, the acronym CIIPX is still listed in the OMIM database as a “neuronal” form of CIPO (OMIM #300048), where omitting of the word “neuronal” would more accurately reflect the observed pathology and genetic findings.

In conclusion, our study broadens the phenotypic spectrum of filaminopathies by highlighting the selective impairment of the gastrointestinal tract due to a novel genetic variant located on the long isoform of *FLNA*, and expands our understanding on the pathogenetic mechanisms that may underlie late-onset genetic CIPO.

## Data Availability

The datasets presented in this study can be found in online repositories. The names of the repository/repositories and accession number(s) can be found below: http://10.5281/zenodo.14421914.14421914.
