# Supplementary material for: A unique case of late-onset CIPO caused by a missense mutation in the long isoform of FLNA
Source: Front Genet. 2025 Aug 8;16:1611614. doi: 10.3389/fgene.2025.1611614 (PMC12370735; doi:10.3389/fgene.2025.1611614)
Supplement: Supplementary file 1 [file DataSheet1.pdf]

Supplementary Material

Figure S1: Pedigree of the family analyzed

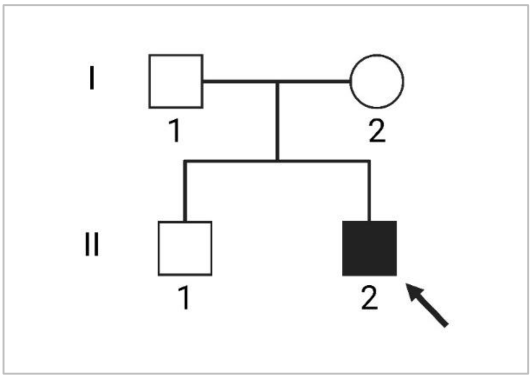

Figure S2: Multiple Sequence Alignment Analysis of the first 60 amino acids of filamin A.

Figure S2: **Multiple Sequence Alignment Analysis of the first 60 amino acids of filamin A.** The mutated glycine in position 19 (Gly19Val) is reported in bold, indicated by the black arrow.

```
                                    ▼
H. Sapiens      MSSSHSRAGQSAAGAAPGGGVDTRDAEMPATEKDLAEDAPWKKIQQNTFTRWCNEHLKCV   60
Gorilla         MSSSHSRAGQSAAGAAPGGGVDTRDAEMPATEKDLAEDAPWKKIQQNTFTRWCNEHLKCV   60
Orangutan       MSSSHSRAGQSAAGAAPGGGVDTRDAEMPATEKDLAEDAPWKKIQQNTFTRWCNEHLKCV   60
Macaca          MSSSHSRAGQSAAGAAPGGGADTRDAEMPATEKDLAEDAPWKKIQQNTFTRWCNEHLKCV   60
Bear            MSSSHSRAGQSAAGAAPGGGTDTRDAEMPATEKDLAEDAPWKKIQQNTFTRWCNEHLKCV   60
Lion            MSSSHSRAGQSAAGAAPGGGTDTRDAEMPATEKDLAEDAPWKKIQQNTFTRWCNEHLKCV   60
Puma            MSSSHSRAGQSAAGAAPGGGTDTRDAEMPATEKDLAEDAPWKKIQQNTFTRWCNEHLKCV   60
Dog             MSSSHSRAGQSAAGAAPGGGADTRDAEMPATEKDLAEDAPWKKIQQNTFTRWCNEHLKCV   60
Mouse           MSSSHSRCGQSAAVASPGGSIDSRDAEMPATEKDLAEDAPWKKIQQNTFTRWCNEHLKCV   60
Rat             MSSSHSRSGQSAAGASPAGGIDTRDAEMPATEKDLAEDAPWKKIQQNTFTRWCNEHLKCV   60
Cavia           MSSFHSRSGQSAAGAAPGGGTDARDAEMPATEKDLAEDAPWKKIQQNTFTRWCNEHLKCV   60
Bos             MSSSHSRAGQSAAGAVLGIGADTRDAEMPATEKDLAEDAPWKKIQQNTFTRWCNEHLKCV   60
```

Figure S3: The hydropathy of the N-terminal of filamin A

The coloration reflects aminoacidic hydropathy, with very hydrophobic amino acids in red to very hydrophilic in blue. In the second line the mutated (Gly19Val) human filamin A is reported, for comparison. As shown by homogeneous colour, the Glycine residue in position 19 (squared) is moderately hydrophilic, whereas Valine is strongly hydrophobic.

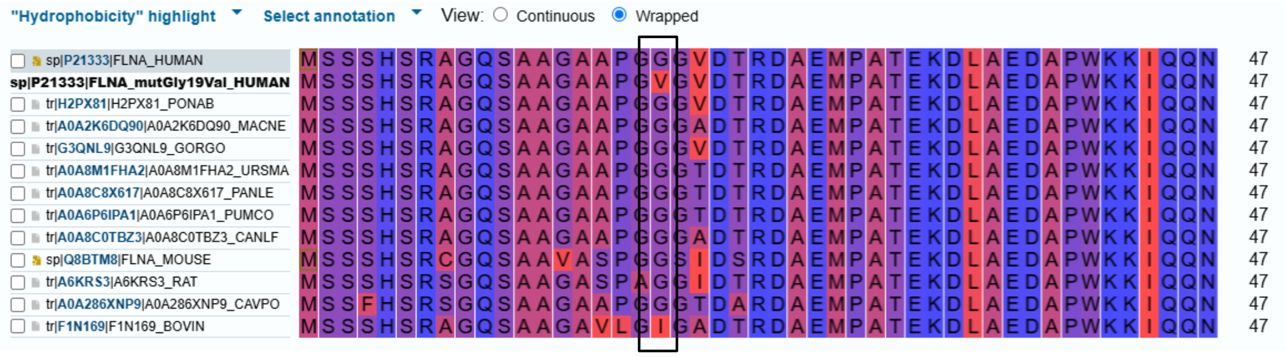

Figure S4: Helix propensity of the filamin A N-terminal

The helix propensity of an amino acid refers to its tendency to form alpha-helices, a common secondary structure in proteins. This propensity is influenced by various factors, including the amino acid side chain properties and its ability to participate in hydrogen bonding within the helix (Pace and Scholtz, 1998). The residues are colored according to their Chou-Fasman helix propensity (Chou and Fasman, 1978). The highest propensity is magenta, the lowest is green. In the second line the mutated (Gly19Val, in the square) human filamin A is reported. Valine has higher propensity to participate in H-bonds compared to glycine; this could have an impact on the secondary structure.

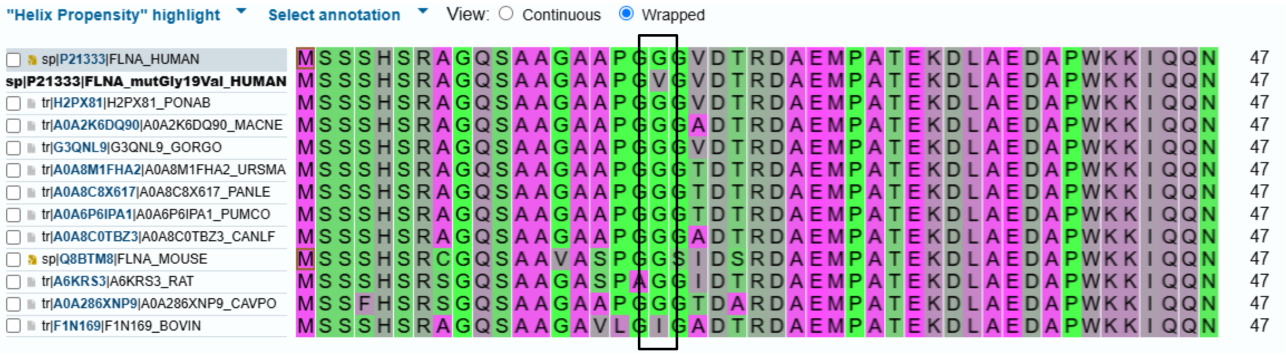

Figure S5: Strand propensity of the filamin A N-terminal

The strand propensity of an amino acid refers to its tendency to form beta-strands, another common secondary structure in proteins (Street and Mayo, 1999). Like helix propensity, strand propensity is influenced by the amino acid side chain properties and its ability to participate in hydrogen bonding within the strand. By understanding the helix and strand propensities of amino acids, it is possible to gain insights into protein folding, stability, and function. The residues are colored according to their Chou-Fasman Strand propensity (Chou and Fasman, 1978). The highest propensity is yellow, the lowest is blue. As observable in the second line, the mutated residues (Gly19Val, in the square) have divergent propensity to form beta-strands; this could have an impact on the secondary structure.

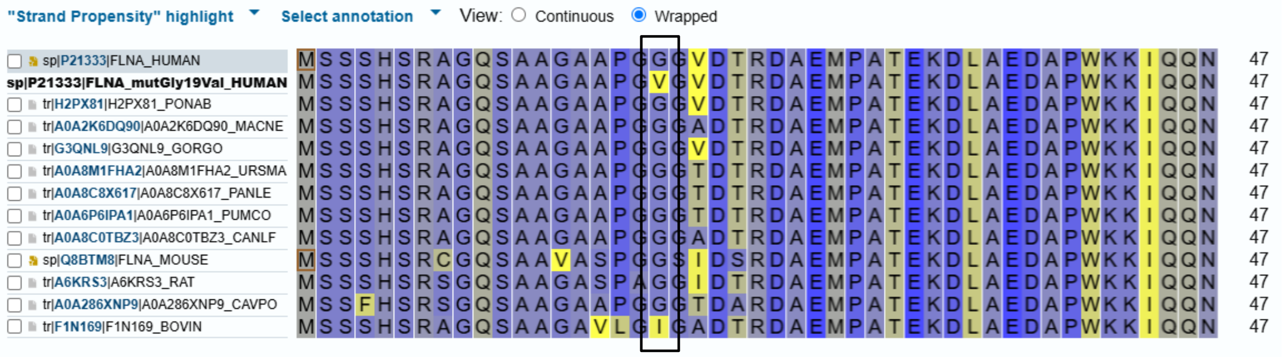

Figure S6: The turn propensity of filamin A N-terminal

The turn propensity of an amino acid refers to its tendency to be found in turns, which are regions of the protein chain that connect secondary structures like alpha helices and beta sheets (Hsu et al., 2006). Turns are characterized by a sharp change in the direction of the polypeptide chain. The residues are colored according to their Chou-Fasman turn propensity (Chou and Fasman, 1978). The highest propensity is red, the lowest is cyan. As observable in the second line, the mutated residues (Gly19Val, in the square) have divergent propensity to be found in turns; this could have an impact on the secondary structure.

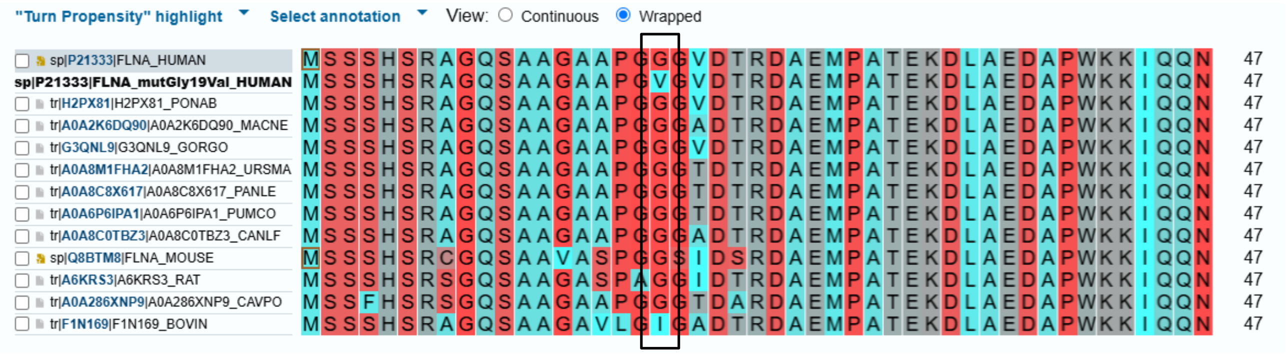

Figure S7: Phylogenetic tree

Phylogenetic representation of the 12 organisms used for alignment, highlighting evolutionary divergence (Consortium, 2025).

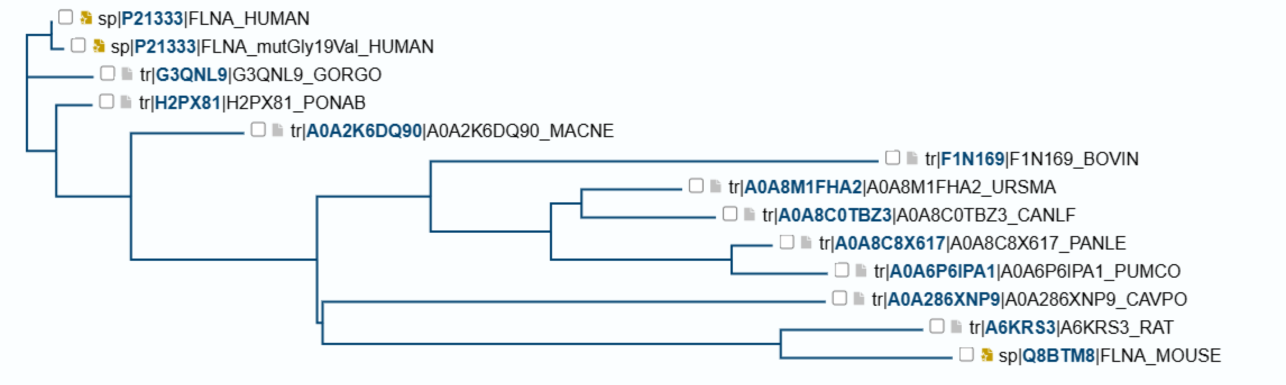

Figure S8: PsiPred prediction of the Actin Binding Domain (ABD) secondary structure [residue 2-274] of Filamin A WT (A) and Gly19Val (B).

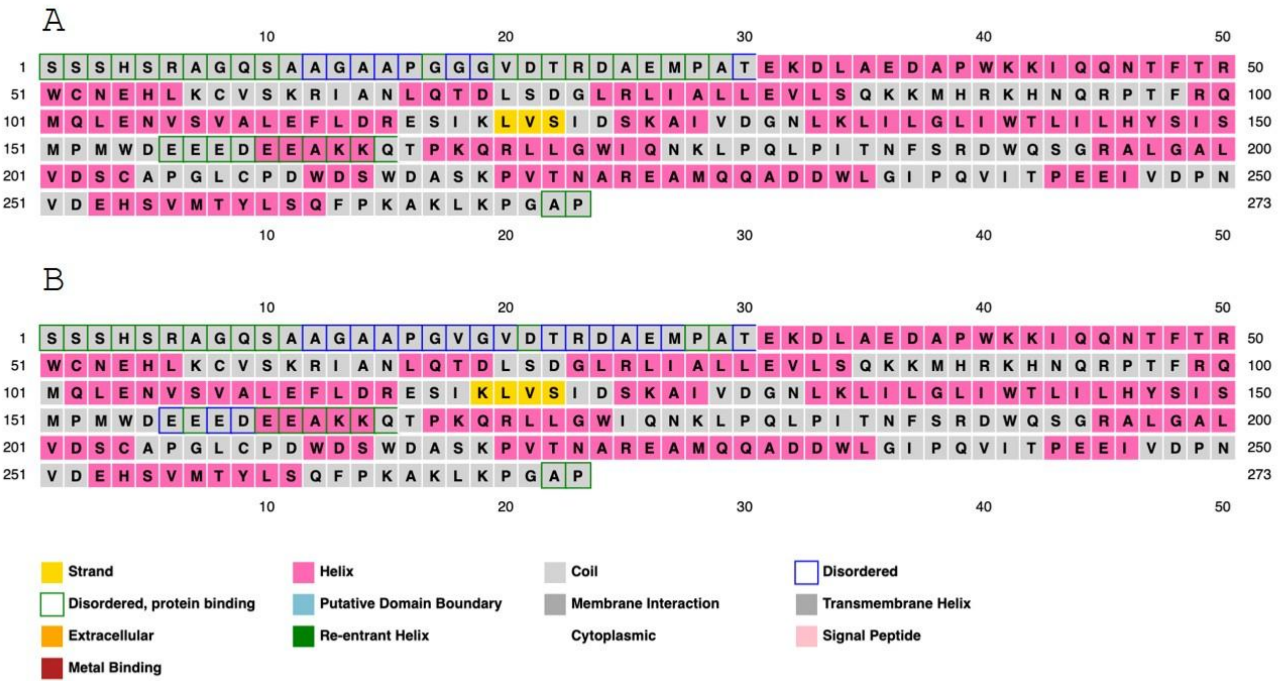

Table S1: Sequences of the oligonucleotides designed for PCR and Sanger Sequencing

	Sequence	T_m	Length
**FLNA EX2F**	5'- CCTAGGTGCCTGCGACTTTA -3'	64°C	548bp
**FLNA EX2R**	5'- GAAGGGGGTGGTTTGGAGG -3'		

Table S2: Touchdown thermal protocol of the FLNA exon 2 PCR

Temperature	Time	
95°C	2 min	
95°C	1 min	20 cycles
65°C – 55°C (-0,5°C/cycle)	1 min	
72°C	30 sec	
95°C	1 min	20 cycles
55°C	1 min	
72°C	30 sec	
4°C	∞	

Table S3: Correspondence between UniProt nomenclature, animal species, and feeding habits (Consortium, 2025)

UniProt FLNA nomenclature	Species	Feeding
sp|P21333|FLNA_HUMAN	Homo sapiens (Human)	omnivores/facultative carnivores
sp|P21333|FLNA_mutGly19Val_HUMAN	Homo sapiens (with mutation Gly19Val)	omnivores/facultative carnivores
tr|H2PX81|H2PX81_PONAB	Pongo abelii (orangutan)	omnivores/facultative carnivores
tr|A0A2K6DQ90|A0A2K6DQ90_MACNE	Macaca nemestrina	omnivores/facultative carnivores
tr|G3QNL9|G3QNL9_GORGO	Gorilla gorilla gorilla	omnivores/facultative carnivores
tr|A0A8M1FHA2|A0A8M1FHA2_URSMA	Ursus maritimus (Polar bear)	omnivores/facultative carnivores
tr|A0A8C8X617|A0A8C8X617_PANLE	Panthera leo (Lion)	obligate carnivores
tr|A0A6P6IPA1|A0A6P6IPA1_PUMCO	Puma concolor (Mountain lion)	obligate carnivores
tr|A0A8C0TBZ3|A0A8C0TBZ3_CANLF	Canis lupus familiaris (Dog)	omnivores/facultative carnivores
sp|Q8BTM8|FLNA_MOUSE	Mus musculus (Mouse)	rodents
tr|A6KRS3|A6KRS3_RAT	Rattus norvegicus (Rat)	rodents
tr|A0A286XNP9|A0A286XNP9_CAVPO	Cavia porcellus (Guinea pig)	rodents
tr|F1N169|F1N169_BOVIN	Bos taurus (Bovine)	ruminants

Supplementary References

Chou, P. Y., and Fasman, G. D. (1978). Empirical predictions of protein conformation. *Annu Rev Biochem* 47, 251–276. doi: 10.1146/ANNUREV.BI.47.070178.001343

Consortium, T. U. (2025). UniProt: the Universal Protein Knowledgebase in 2025. *Nucleic Acids Res* 53, D609–D617. doi: 10.1093/nar/gkae1010

Hsu, H. J., Chang, H. J., Peng, H. P., Huang, S. S., Lin, M. Y., and Yang, A. S. (2006). Assessing computational amino acid beta-turn propensities with a phage-displayed combinatorial library and directed evolution. *Structure* 14, 1499–1510. doi: 10.1016/J.STR.2006.08.006

Pace, C. N., and Scholtz, J. M. (1998). A helix propensity scale based on experimental studies of peptides and proteins. *Biophys J* 75, 422. doi: 10.1016/S0006-3495(98)77529-0

Street, A. G., and Mayo, S. L. (1999). Intrinsic β-sheet propensities result from van der Waals interactions between side chains and the local backbone. *Proc Natl Acad Sci U S A* 96, 9074–9076. doi: 10.1073/PNAS.96.16.9074/ASSET/31F8DD3C-760F-4299-96CA-20E6879FD7A1/ASSETS/GRAPHIC/PQ1692170001.JPEG
